# Supplementary material for: Expression and Functional Analysis of the Smo Protein in Apis mellifera
Source: Insects. 2024 Jul 22;15(7):555. doi: 10.3390/insects15070555 (PMC11277047; doi:10.3390/insects15070555)
Supplement: Supplementary file 1 [file insects-15-00555-s001.zip › insects-3082048-supplementary.pdf]

The following are the data from the experimental section of this manuscript on the Y-type olfactometer:

Table S1. Selection of different odor substances in bees after feeding cyclopamine

	Control		Cyclopamine		
Odor Selection	Odor Selection	Blank selection	Odor Selection	Blank selection	P
6-methyl-5-heptene-2-one(6-M)	32	18	28	22	0.524
linalool	29	21	6	44	＜0.0001
neral	34	16	23	27	0.006
VUAA1	25	25	14	36	0.001
myrcene	34	16	13	37	＜0.0001

Table S2. Selection of different odor substances in bees after feeding purmorphamine

	Control		Purmorphamine		
Odor Selection	Odor Selection	Blank selection	Odor Selection	Blank selection	P
6-methyl-5-heptene-2-one(6-M)	12	38	23	27	0.002
linalool	16	34	30	20	＜0.0001
neral	17	43	20	30	0.465
VUAA1	24	26	29	21	0.569
myrcene	20	30	21	29	0.886
